# Cellular, Extracellular and Extracellular Vesicular miRNA Profiles of Pre-Ovulatory Follicles Indicate Signaling Disturbances in Polycystic Ovaries

**DOI:** 10.3390/ijms21249550

**Published:** 2020-12-15

**Authors:** Ilmatar Rooda, Mohammad Mehedi Hasan, Kristine Roos, Janeli Viil, Aneta Andronowska, Olli-Pekka Smolander, Ülle Jaakma, Andres Salumets, Alireza Fazeli, Agne Velthut-Meikas

**Affiliations:** 1 Department of Chemistry and Biotechnology, Tallinn University of Technology, Akadeemia tee 15, 12618 Tallinn, Estonia; ilmatar.rooda@gmail.com (I.R.); kristine.roos@gmail.com (K.R.); olli-pekka.smolander@taltech.ee (O.-P.S.); 2 Competence Centre on Health Technologies, Teaduspargi 13, 50411 Tartu, Estonia; andres.salumets@ccht.ee; 3 Department of Pathophysiology, Institute of Biomedicine and Translational Medicine, University of Tartu, Ravila 14B, 50411 Tartu, Estonia; mehedi.hasan@ut.ee (M.M.H.); janeli.viil@ut.ee (J.V.); alireza.fazeli@ut.ee (A.F.); 4 Nova Vita Clinic, A. H. Tammsaare tee 47, 11314 Tallinn, Estonia; 5 Institute of Animal Reproduction and Food Research, Polish Academy of Sciences, Tuwima St. 10, 10-748 Olsztyn, Poland; a.andronowska@pan.olsztyn.pl; 6 Institute of Veterinary Medicine and Animal Sciences, Estonian University of Life Sciences, Fr. R. Kreutzwaldi 1, 51006 Tartu, Estonia; ylle.jaakma@emu.ee; 7 Department of Obstetrics and Gynecology, Institute of Clinical Medicine, University of Tartu, L. Puusepa St. 8, 50406 Tartu, Estonia; 8 Institute of Genomics, University of Tartu, Riia 23b, 51010 Tartu, Estonia; 9 Academic Unit of Reproductive and Developmental Medicine, Department of Oncology and Metabolism, The Medical School, University of Sheffield, Sheffield S10 2SF, UK

**Keywords:** extracellular vesicles, human ovarian follicle, granulosa cells, follicular fluid, polycystic ovary syndrome, PCOS, miRNA, intercellular communication

## Abstract

Cell-free RNAs have the potential to act as a means of gene expression regulation between cells and are therefore used as diagnostic markers describing the state of tissue environment. The origin and functions of such RNAs in human ovarian follicle, the environment of oocyte maturation, are unclear. The current study investigates the difference in the microRNA profiles of fertile women and polycystic ovary syndrome (PCOS) patients in three compartments from the same preovulatory follicle: mural granulosa cells (MGC), cell-free follicular fluid (FF), and extracellular vesicles (EV) of the FF by small RNA sequencing. In silico analysis was used for the prediction and over-representation of targeted pathways for the detected microRNAs. PCOS follicles were distinguished from normal tissue by the differential expression of 30 microRNAs in MGC and 10 microRNAs in FF (FDR < 0.1) that commonly regulate cytokine signaling pathways. The concentration of EV-s was higher in the FF of PCOS patients (*p* = 0.04) containing eight differentially expressed microRNAs (*p* < 0.05). In addition, we present the microRNA profiles of MGC, FF, and EV in the fertile follicle and demonstrate that microRNAs loaded into EVs target mRNAs of distinct signaling pathways in comparison to microRNAs in FF. To conclude, the three follicular compartments play distinct roles in the signaling disturbances associated with PCOS.

## 1. Introduction

Disturbances in normal ovarian physiology cause subfertility or infertility leading to prolonged effort or inability for a woman to conceive. Polycystic ovary syndrome (PCOS) is a common hormonal disturbance affecting up to 20% reproductive age women worldwide [1]. PCOS is a complex syndrome with reproductive, metabolic and psychological features and is characterized by hyperandrogenism, obesity, insulin resistance, polycystic ovarian morphology (PCOM) and/or anovulation [2,3]. The phenotype varies broadly depending on the genotype, ethnicity, and environmental factors [1]. Heterogeneity of PCOS is a challenge for diagnostics causing delayed detection and dissatisfaction with care [4]. The current knowledge regarding molecular mechanisms behind the dysfunction of PCOS is still incomplete.

The human ovarian follicle is a dynamic structure that supports oocyte maturation, ovulation, and steroid hormone synthesis. Granulosa, theca, and follicular immune cells are the somatic cell populations ensuring the flawless performance of the above-mentioned processes crucial for female fertility. By the pre-ovulatory stage, the follicle diameter expands above 20 mm and is filled with follicular fluid (FF) [5]. This fluid-filled environment enables long-distance cell communication between different cell populations via cell-secreted (lipo)proteins, ribo-protein complexes (RBPs), and extracellular vesicles (EVs) containing nucleic acids and proteins from the secreting cells [6]. The possible disorders in long-distance intercellular signaling in human polycystic ovaries have not been thoroughly investigated.

EVs are lipid bilayer-coated nanoparticles in varying size range [7]. Based on their size and mode of biogenesis EVs are classified into three major subtypes; exosomes (40–100 nm), microvesicles (100–500 nm), and apoptotic bodies (500 nm–2 um) [8]. The release of EVs and RBPs has been extensively studied and attributed to all cell types in the human body. Moreover, cell-free RNAs in RBPs and EVs have been detected in all investigated body fluids, including FF [9,10]. Small RNAs in EVs have caught more attention, however more than 90% of circulating miRNAs are present outside of EVs associated with AGO2, nucleophosmin 1, or high-density lipoprotein, among other proteins [6]. The secretion of RNA molecules via EVs is at least partly controlled by the releasing cells and the RNA content of EVs is cell specific [11]. However, AGO2-miRNA complexes may also be released non-specifically into extracellular space following cell death [6]. The RNA content of both EVs and RBPs can be taken up by recipient cells from the same or another cell population and potentially modulates signaling pathways in the recipient [12]. Examples of significance of such long-distance communication can be drawn from cancer studies and immunology [13], among other fields. In reproductive studies, fluorescently labelled EVs isolated from FF were taken up by ovarian granulosa cells in an equine in vitro model, suggesting that the exchange of RNA is potentially an important mean of communication also in the normal ovarian physiology [14]. Up to now, mainly miRNAs have been widely studied as the constituents of the follicular EVs due to their well-known molecular function. However, also other types of long and small RNAs have been described as the components of EVs and RBPs: mRNA, lncRNA, SRP RNA, circRNA, snRNA, snoRNA, vault RNA, Y RNA, piRNA, tRNA, and rRNA fragments [6]. The extracellular RNA content has been proposed as a diagnostic tool for disease states, as several cell populations have been demonstrated to change the repertoire of released cell-free RNAs upon external stimulus or disease [15,16].

The current study hypothesizes that there are differences in the cellular and extracellular miRNA expression levels between the ovarian follicles of healthy and PCOS patients indicating molecular signaling disturbances at preovulatory stage. We set out to investigate in a genome-wide manner the cellular and extracellular miRNA profile of three matched sample types collected from single follicles of healthy fertile women versus PCOS patients comprising of granulosa cells (MGC), cell-depleted FF and EVs purified from the FF. Such combined dataset for the human follicle is unique. The obtained information will provide new avenues for therapeutic approaches for PCOS patients, e.g., the development of new ovarian stimulation as well as in vitro oocyte maturation protocols for improving their infertility treatment outcomes.

## 2. Results

**Figure 1:**
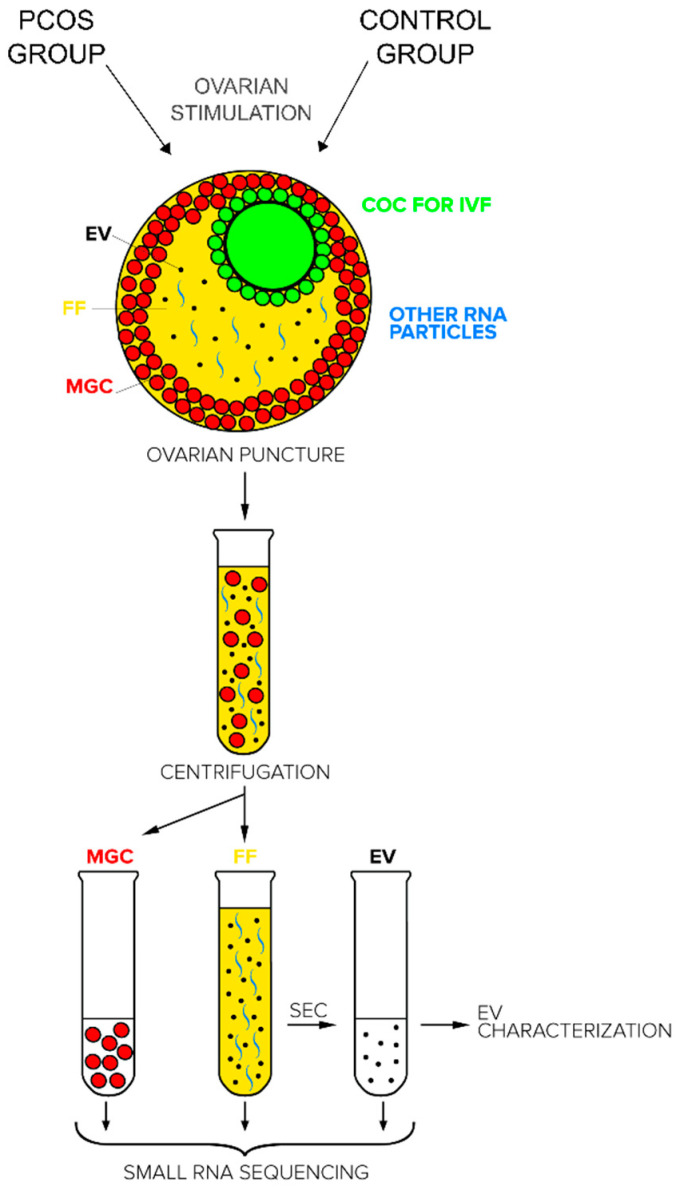
Schematic description of sample collection and processing from human pre-ovulatory follicles of polycystic ovary syndrome (PCOS) patients and fertile control group. COC—cumulus oocyte complex (green), FF—cell-free follicular fluid (yellow) containing extracellular vesicles (EV, black) and non-EV-bound RNA (blue), IVF—in vitro fertilization, MGC—mural granulosa cells (red).

Intrafollicular communication differences between the fertile and PCOS ovaries were modelled by analyzing material from three distinct sources, each collected from the same follicle: small RNA from MGC, all cell-free small RNA populations from FF, and small RNA in EVs purified from the FF. The rationale of sample collection and compartmentalization is outlined in Figure 1.

EVs were characterized for their size, concentration, and surface markers. All three sample types underwent small RNA sequencing, and the results were validated by real-time quantitative PCR (RT-qPCR). The main characteristics of patients and the number of analyzed samples according to each method are presented in Table 1.

### 2.1. Characterization of Nanoparticles Isolated from Human Follicular Fluid as Extracellular Vesicles

**Figure 2:**
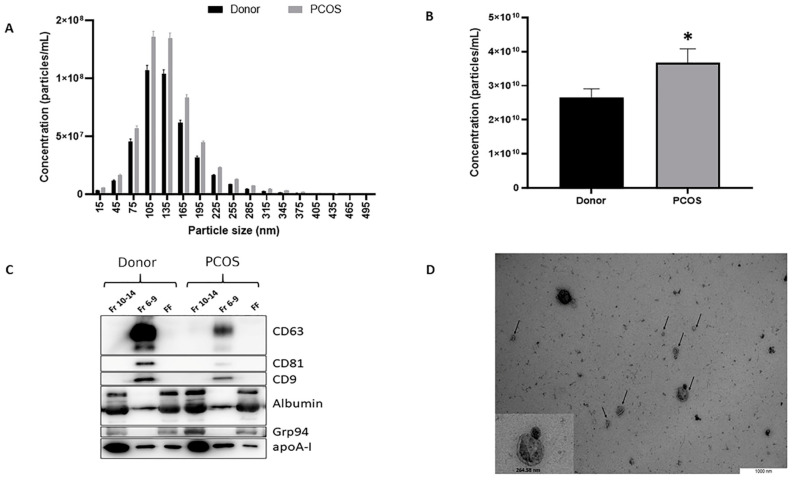
Characterization of extracellular vesicles (EVs) isolated from cell-free follicular fluid (FF). (**A**) Size profile of EVs in study groups (mean ± SEM). (**B**) Concentration of EVs in polycystic ovarian syndrome patients (PCOS) and control samples (mean ±SEM). (**C**) Positive signals of EV markers CD63, CD81, CD9 were detected from EV samples (Fr 6-9), while undetectable from protein fractions (Fr 10-14) and FF samples before EV isolation (FF). Albumin, Grp94, and apoA-I were used as markers of negative selection demonstrating diminished signal intensity in EV samples compared to the protein fraction and FF samples. (**D**) Transmission electron microscopy analysis of purified EVs, indicated by arrows. Data in (**A**,**B**) is demonstrated as mean ± SEM. * *p* = 0.04 Student’s *t*-test.

FF-derived EVs from SEC fractions 6–9 (Supplementary Figure S1) were characterized by three independent methods: NTA, TEM, and Western blot (WB) analysis (Figure 2). According to the NTA size profile analysis (Figure 2A), most of the nanoparticles were under 200 nm in diameter with a large population range within 75–165 nm, which is a typical EV size range [17]. We observed a 4-nm difference between healthy women (mean 138.6± SEM 0.2 nm) and PCOS patients (142.7 ± 0.2 nm) FF-derived EVs (*p* < 2.2 × 10^−16^, Supplementary Figure S2). Secondly, the PCOS FF samples contain higher EV concentration compared to the control group (*p* = 0.04, Figure 2B).

Tetraspanins CD63, CD81, and CD9, considered as positive EV markers, were used to verify the presence and enrichment of extracellular vesicles in EV preparations. Based on WB analysis (Figure 2C), all tetraspanins were enriched in EVs, whereas the protein fractions of FF and non-purified FF had undetectable levels of the studied EV markers. Endoplasmic reticulum protein Grp94, which is expected to be absent or under-represented in smaller EVs, notably exosomes [18], was indeed absent from EV samples, whereas protein fractions and non-purified FF were positive for Grp94. The purity of EVs and the efficiency of SEC was also tested by analyzing the presence of albumin and apolipoprotein A-I (apoA-I) as these proteins are often co-isolated with EVs. Strong signals were detected in FF samples, and although both of these proteins were also detectable in EV samples, the largest quantities were enriched in later fractions (Fr 10–14) corresponding to protein enrichment (Figure 2C). TEM analysis also confirmed the presence of EVs in the studied samples (Figure 2D).

### 2.2. Small RNA Profile of Granulosa Cells, Cell-Free Follicular Fluid and Extracellular Vesicles

Whole-genome small RNA sequencing was performed for all three sample types (MGC, FF and EV) to model intrafollicular signaling for eight oocyte donors and seven PCOS patients (Table 1). The sequencing depth and mapping efficiency for each sample type are represented in Supplementary Table S1. Analysis of sequencing read size distribution after adapter trimming demonstrated different patterns of small RNA sequence lengths between sample types (Supplementary Figure S3) referring to distinct variability in their content of small RNA populations. A prevalent size peak at 17–24 nt corresponding to the length of miRNAs appears in all samples. In addition, MGC and FF samples contain small RNAs of 28–36 nt length not detected in EV samples. Average sequence length is also shorter in EV samples compared to FF and MGC samples (*p* < 0.005, Supplementary Table S1). The current study focuses further on the miRNA content of each sample type.

### 2.3. miRNAs in Granulosa Cells, Cell-Free Follicular Fluid and Extracellular Vesicles

In total 1525 unique miRNAs were detected by at least one read: 658 miRNAs were observed in EVs, 1060 in FFs and 1381 in MGCs. All sample types share a large proportion of the most abundant miRNAs among the top 20 most represented sequences in every sample type (EV and FF share 15, FF and MGC share eight out of 20, Supplementary Table S2).

**Figure 3:**
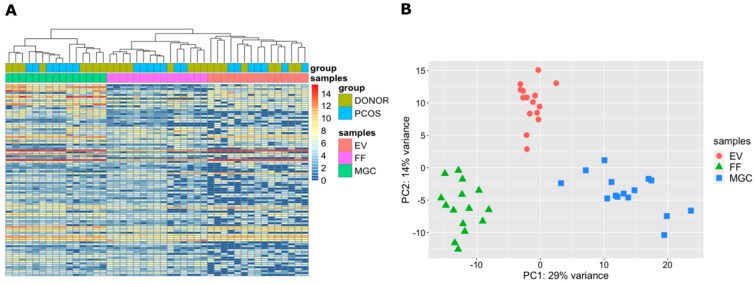
Cluster analysis of investigated samples and study groups. (**A**) Hierarchical clustering of top 100 most variable miRNAs across all samples. Results are depicted by DESeq2 normalized counts on log_2_ scale. (**B**) Principal component analysis based on expressed miRNAs per sample type. EV—extracellular vesicles, FF—cell-free follicular fluid, MGC—mural granulosa cells, PCOS—polycystic ovary syndrome.

Clustering analysis revealed significant differences between sample types according to their miRNA content (Figure 3A,B). As expected, EV and FF samples cluster closer to each other in comparison to MGC samples (Figure 3A), as EV samples are a sub-compartment purified from the corresponding FF samples.

### 2.4. Cellular and Extracellular miRNAs in the Healthy Ovarian Follicle

**Figure 4:**
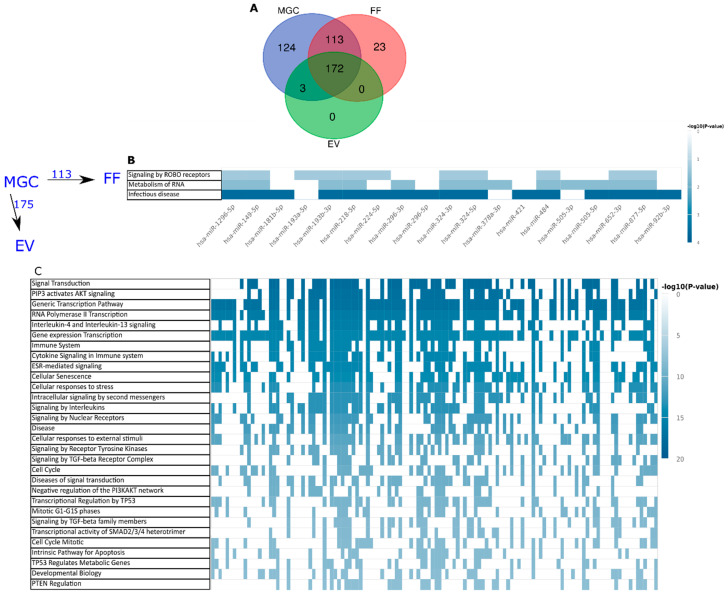
Cellular and extracellular miRNAs observed in ovarian follicles of fertile women. (**A**) Distribution of observed miRNAs (>5 reads in >50% of samples) between sample types. (**B**) Pathways over-represented by the targets of 113 miRNAs secreted by mural granulosa cells (MGC) into follicular fluid (FF) outside of extracellular vesicles (EV). (**C**) Top 30 pathways potentially regulated by 175 miRNAs secreted into FF in EVs. Each column in (**B**,**C**) corresponds to one miRNA regulating a pathway, if marked in blue.

The analysis of samples from oocyte donors (*n* = 8) representing the healthy ovary revealed that in total 172 miRNAs are common to all three sample types (>5 reads observed in >50% of samples per sample type). A set of 124 miRNAs were only present in MGC samples indicating that these are not secreted out of the cells (Figure 4A, Supplementary Table S3).

EVs did not contain any unique miRNAs compared to FF or MGC. Three miRNAs (hsa-miR-374a-5p, hsa-miR-190a-5p and hsa-miR-196a-5p) were shared only between EV and MGC samples and not present in FF. We hypothesize that these miRNAs are specifically enriched into EVs. In the FF samples that contain RNA molecules present also in other forms these miRNAs remain below detection limit.

Moreover, MGC samples share 285 miRNAs with FF. Twenty-three miRNAs are exclusive to FF samples. Comparison to a recent study [19] analyzing female serum and plasma miRNAs extracted with the same protocol as in the current paper revealed that 10 of the miRNAs observed uniquely in FF potentially derive from plasma infiltrating into the follicle from perifollicular capillaries (Supplementary Table S3).

A list of 175 miRNAs were detected in EVs indicating that these miRNAs are secreted into the follicular space in vesicular form. At the same time, 113 miRNAs were common to MGC and FF only (Figure 4A), suggesting that these are secreted from cells in complexes other than EVs with characteristic surface markers. By comparing these two lists we were interested to test, if the secretion of miRNAs in EVs serves a different signaling purpose in comparison to other mechanisms of miRNA secretion. We investigated, if the miRNAs inside or outside of EVs have a potential to regulate overlapping signaling pathways. Indeed, only three pathways were commonly targeted by 11–16 FF miRNAs residing in FF outside of EVs (Figure 4B, Supplementary Table S4A). On the other hand, 436 pathways were over-represented for miRNAs in EVs, the top 30 of the pathways were common to 30-94 miRNAs (Figure 4C, Supplementary Table S4B). The 23 miRNAs observed only in FF were omitted from the pathway over-representation analysis, as these are not probably secreted by MGC as mentioned above.

**Figure 5:**
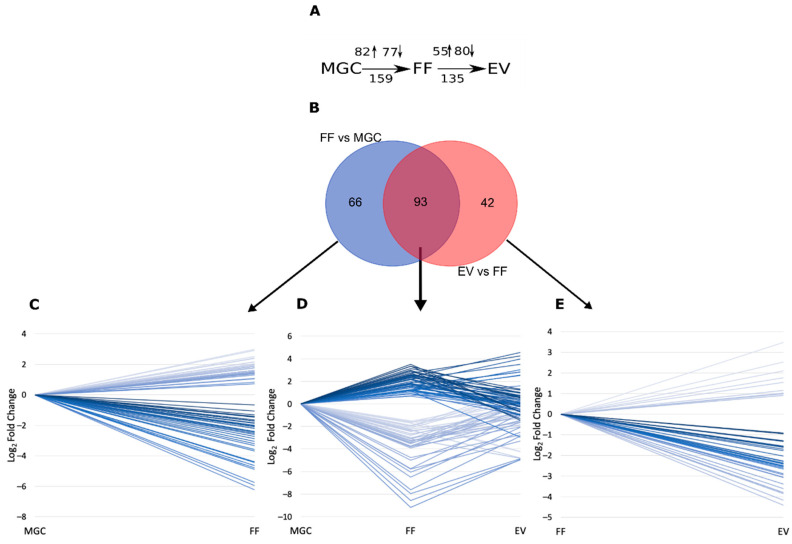
Comparison of cellular and cell-free miRNA expression levels. (**A**) Schematic representation of miRNA expression level comparisons performed between sample types and the number of differentially expressed (DE) results obtained. Arrows depict the number of upregulated (↑) or downregulated (↓) genes in each comparison. (**B**) Number of distinct and shared DE miRNAs between comparisons. (**C**) DE results unique to FF vs. MGC comparisons (**D**) DE results common for FF vs. MGC and EV vs. FF comparisons. (**E**) DE results unique to EV vs. FF comparisons. Each line in panels (**C**–**E**) depicts the statistically significant (FDR < 0.05) expression level difference between sample types of one miRNA. EV—extracellular vesicles, FF—cell-free follicular fluid, MGC – mural granulosa cells.

Significant enrichment depicting RNA secretion from cells to FF and the difference between miRNAs loaded into EVs in comparison to all cell-free miRNAs was analyzed by differential expression (DE) analysis in control group samples (FF vs. MGC and EV vs. FF, respectively, as depicted in Figure 5A).

Comparison between FF and MGC samples, resulted in 159 differentially expressed miRNAs (FDR < 0.05, Figure 5A, Supplementary Figure S4A, Supplementary Table S5), while the DE analysis of EV and FF samples demonstrated the statistically significant expression of 135 miRNAs (FDR < 0.05, Figure 5A, Supplementary Figure S4B, Supplementary Table S6). From all differentially expressed miRNAs 93 were common to both comparisons: FF vs. MGC and EV vs. FF (Figure 5B). Figure 5D illustrates the expression level changes of those 93 miRNAs throughout MGC, FF and EV samples. Figure 5C and Figure 5E illustrates miRNA expression changes of 66 and 42 miRNAs differentially expressed between FF vs. MGC and EV vs. FF, respectively. Significant miRNA expression changes between the analyzed compartments indicate potentially different secretion mechanisms: miRNAs with constantly increasing levels from MGC to FF and EV are more probably packed specifically into EVs. In contrast, miRNAs with the highest abundance in FF have a higher probability to be secreted into extracellular space in other macromolecular complexes.

### 2.5. Differences in miRNA Expression between PCOS Patients and Oocyte Donors

**Figure 6:**
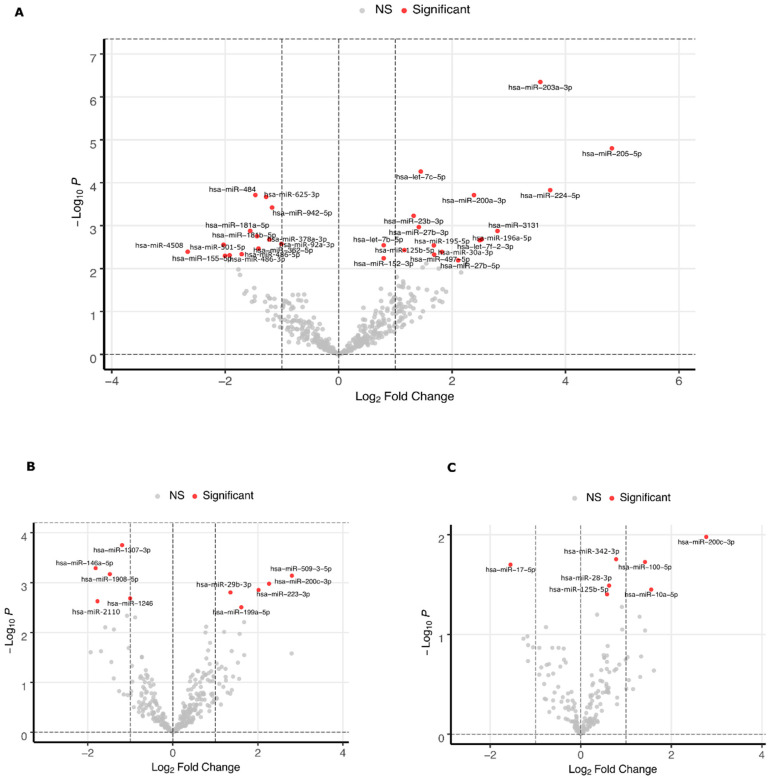
Differentially expressed miRNAs between oocyte donors and PCOS patients in mural granulosa cell samples (**A**), cell-free follicular fluid samples (**B**) and extracellular vesicle samples **(C)**. Statistical significance cut-off is FDR < 0.1 (A and B) or *p* < 0.05 (**C**).

A comparison of samples between patient groups revealed that 30 and 10 miRNAs in MGC and FF, respectively, were differentially expressed between PCOS and oocyte donor patients (FDR <0.1, Figure 6A,B, Supplementary Table S7A,B). Due to a higher variation of miRNA expression levels across patients in EV samples (Figure 3A), no miRNAs reached the same FDR cut-off level. However, seven miRNAs were differentially expressed in EV samples between the two groups without considering the FDR (*p*-value < 0.05, Figure 6C, Supplementary Table S7C). Hsa-miR-200c-3p was the only commonly up-regulated miRNA in the extracellular samples FF and EV. All other DE miRNAs between the patient groups were unique to each sample type.

Validation of the RNA sequencing results was performed by RT-qPCR for miRNAs with the highest fold change and that have been previously related to ovarian functions (Table 2). Since oocyte donors are generally young women the average age difference between the two study groups in RNA sequencing experiment was statistically significant (*p*-value = 0.002, Table 1). As there is previous evidence that the expression of some miRNAs can be affected by age [20,21], a validation cohort of age-matching women undergoing IVF due to male-factor infertility was added to the oocyte donor samples used for RNA sequencing. The average age difference between the PCOS and RT-qPCR validation control group was therefore not statistically significantly (*p*-value = 0.626, Table 1). From five of the validated miRNAs in MGC samples the expression change direction was confirmed with statistical significance for hsa-let-7c-5p, hsa-miR-196a-5p and hsa-miR-203-3p. In FF samples four of the five validated miRNAs presented same directional change, out of which two (miRNAs hsa-miR-509-3-5p and has-miR-200c-3p) were also statistically significant. All three validated miRNAs from EVs displayed the same directional change without reaching the statistically significant level (Supplementary Figure S5).

### 2.6. Distinctive Functions Are Dysregulated in Each Analyzed Follicular Compartment of PCOS Patients

As one miRNA may target several genes and one gene may be targeted by several miRNAs, we subsequently aimed to detect any common pathways changed in PCOS patients by the differentially expressed miRNAs. Reactome pathway over-representation was performed for all differentially expressed miRNA-s according to RNA sequencing in each sample type. Lists demonstrating higher and lower miRNA expression levels in PCOS group compared to controls were analyzed separately.

**Figure 7:**
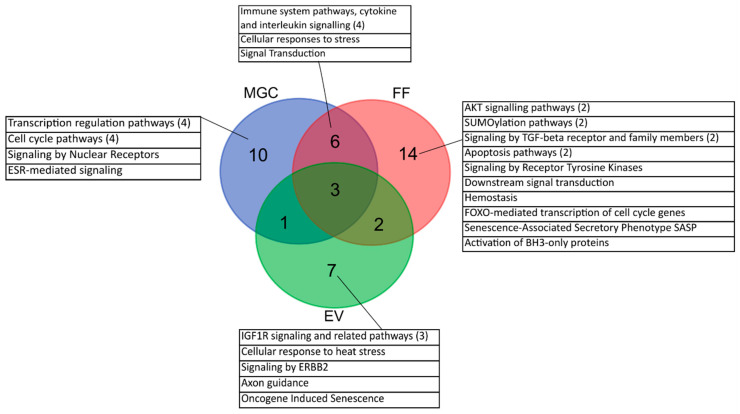
Number of Reactome pathways over-represented for miRNAs that are more abundantly expressed in PCOS patients compared to the fertile control group in each sample type. Numbers in brackets refer to combined pathways with similar outcome. EV—extracellular vesicles, FF—cell-free follicular fluid, MGC—mural granulosa cells.

miRNAs that are significantly upregulated in the PCOS group regulate in total 20 pathways in MGC, 25 pathways in FF and 13 pathways in EV (Figure 7, Supplementary Table S8). Transcription regulation and cell cycle pathways constitute the majority of the over-represented terms in MGC. In addition, miRNAs regulating signaling by estrogen receptors (ESR) and nuclear receptors in general were more abundant in the MGC of PCOS patients.

Over-represented miRNAs in FF regulate several signal transduction pathways: AKT, TGF-beta, as well as pathways related to apoptosis and protein modification by SUMOylation appeared as the most frequent terms. The most common terms for miRNA-s that were significantly more abundant in the EVs of PCOS women were related to IGF1R signaling pathways.

In addition, miRNAs up-regulated in the MGC and FF samples of PCOS women target common immune system related pathways that are not apparent predicted targets for miRNAs in the EVs.

miRNAs that were less abundant in the MGC of PCOS patients are involved in pathway “Cellular responses to external stimuli”. No over-representation of pathways was achieved for miRNAs with low abundance in FF and EV in the PCOS group (Supplementary Table S8).

### 2.7. Potential Novel miRNA as Marker for Follicular EVs

**Figure 8:**
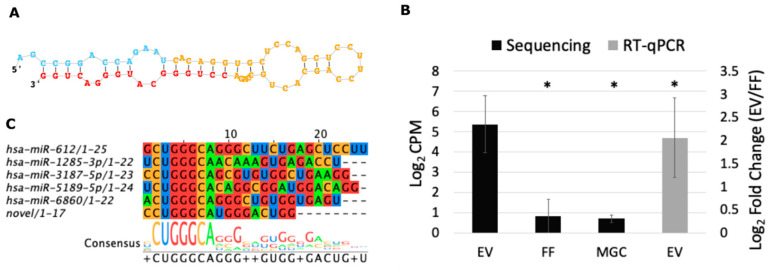
Novel miRNA detected by small RNA sequencing of single follicle components. (**A**) Predicted stem-loop sequence by miRDeep2. (**B**) Novel miRNA expression levels in the extracellular vesicles of follicular fluid (EV), cell-free follicular fluid (FF) and in granulosa cells (MGC) according to RNA sequencing displayed as a mean of count per million (CPM) ±SEM on log_2_ scale (*n* = 15). RT-qPCR validation result is displayed as fold change ±SEM on log_2_ scale (*n* = 15). (**C**) Novel miRNA aligned to previously annotated miRNAs with similar seed sequence. * *p* < 0.05, Student’s t-test.

RNA sequencing data can be useful for predicting novel, yet unannotated miRNAs. After filtering candidate novel miRNA sequences suggested by miRDeep2 algorithm, we propose one potential new miRNA (mature sequence: CCUGGGCAUGGGACUGG, predicted stem-loop sequence in Figure 8A) that was expressed in 12 different patients and in all three sample types (EV, FF and MGC). It was most frequently detected in EV samples (in nine EV samples, four FF, and five MGC samples) demonstrating significantly higher expression levels compared to FF and MGC samples (Figure 8B). Moreover, validation with RT-qPCR demonstrated significantly higher levels of this sequence in EVs compared to FF samples (Figure 8B). The expression levels of the novel miRNA did not differ between PCOS and donor group in any of the sample types (data not shown). Five previously annotated miRNAs share a similar seed sequence (nucleotides 2–8 from 5′ end) with the potential novel miRNA (Figure 8C). miRDB predicted 1430 potential targets for the novel miRNA that were further enriched into 82 terms in the ontology domain of biological processes (FDR < 0.05, Supplementary Table S9). These were further reduced by semantic similarity analysis to three largest categories: regulation of cell communication, cell junction organization, and nervous system development (Supplementary Figure S6).

## 3. Discussion

A key aspect of cellular and organismal homeostasis in the higher mammals is intercellular communication, where cells are required to communicate with each other in order to maintain the vital functions of the body. Some of the important mediators of this cell-cell communication are cell-free RBPs and nanoparticles, including EVs that contain molecules from a plethora of RNA biotypes [6]. All body fluids, including the follicular fluid, are rich sources of cell-free nucleic acids and EVs [39]. EVs are heterogeneous, and their subtypes; exosome and microvesicles share a similar size range, which imposes a challenge in their efficient isolation, purification, and separation [40]. There are several different methods used for EVs isolation, and every method has its own limitations towards the purity of EVs from other RNA-containing particles. According to the Minimal Information for Studies of Extracellular Vesicles 2018 guidelines (MISEV) [39], purification methods should be chosen based on the downstream application of EVs. In the current study the MISEV-approved SEC method that gives the flexibility to separate the EVs according to their size ranges was used to acquire pure and functional EVs with reasonable recovery rates [41,42,43].

Several studies suggest that EVs are involved in intercellular communication in both normal physiology and pathological condition [44]. A recent study presented the higher blood plasma concentration of platelet-derived microparticles in PCOS patients as compared to healthy women [45]. Our study additionally detected significantly more EVs in the FF of PCOS women compared to oocyte donors with normal ovarian morphology.

We have, for the first time, analyzed matched cellular and extracellular small RNA profiles from individual pre-ovulatory follicles by whole-genome small RNA sequencing. In addition, the extracellular small RNAs were analyzed in two fractions: small RNAs loaded into EVs and total cell-free small RNAs present in FF. While separate RNA sequencing studies of MGC [27], FF [9] and EV [9] samples from the human follicle are available, combining the data from the three compartments of the follicle as a closed biological system is unique to the current study. The functionality of extracellular RNAs can roughly be divided into three: known function, predicted function and unknown function [46]. MiRNAs belong into the known function group and therefore we continued to investigate the miRNA profiles of the samples. We observed that the miRNA profile clearly distinguishes the three sample types indicating that the miRNAs are released in EV- and non-EV-mediated mechanisms serve distinct functional purposes. To support our results, it has been demonstrated that the cell-free and EV-associated miRNAs also have different profiles in matching plasma samples [47,48].

There are many possible sorting mechanisms proposed for loading miRNAs into EVs: sequence characteristics, post-transcriptional modifications, subcellular location and intracellular concentration have all been shown to pose an effect to the segregation of miRNAs into EVs [6]. The heterogeneity of EVs (exosomes, microvesicles or apoptotic bodies) additionally plays a role in the nucleic acid content [49]. An even larger proportion of extracellular small RNAs is secreted outside of EVs in composition of other macromolecular complexes, e.g., non-vesicular RBPs. The mechanisms by which proteins interact with miRNAs before secretion remain unclear and may depend on the particular protein in the complex, but the export of miRNAs via these pathways has been shown to be an energy-dependent process [6].

According to our study, 172 miRNAs were present in all sample types, indicating that those miRNAs are non-selectively secreted from cells via both methods (EVs and/or non-vesicular RBP pathway). Moreover, 113 miRNAs were detected only in cells or FF, meaning those miRNAs are likely secreted out of the cells in RBP, while 175 miRNAs were observed also in the EVs. The over-representation analysis of the EV and FF miRNA targets indicate that miRNAs loaded into EVs carry specific molecular signals. It is considered that cargo segregation into EVs is not a random event: for example the human bone marrow- and adipose-mesenchymal stem cell-derived exosomes contain distinctive small RNA molecules linked to their differentiation status [50]. Moreover, studies in the equine and bovine models have demonstrated that follicular fluid EVs were taken up by granulosa cells in vitro and this process affected the expression of genes involved in follicle development [14,51]. Furthermore, some miRNAs in follicular EVs may also regulate oocyte growth, as alterations in their expression were observed between follicles with different oocyte maturation stages [52]. All these studies lead to a conclusion that EVs in follicular fluid most likely have important regulatory roles.

The miRNAs in EV samples potentially target the following Reactome terms: “ESR-mediated signaling”, “Signaling by Nuclear Receptors”, and “PTEN Regulation”, among others. Estrogen receptors belong to the nuclear receptor family and together with other members, like the androgen receptors, are involved in follicle development and ovulation [53,54]. PTEN participates in follicle activation and growth, higher PTEN levels being associated with poor oocyte competence [55,56]. To summarize, miRNA profile analysis of samples from fertile women indicates specific miRNA segregation into vesicles with various targeted pathways downstream. At the same time, the selection of miRNAs for secretion by non-EV mediated pathway tends to be a relatively random event in the follicle.

One may argue whether all the miRNAs detected in extracellular space participate in cell-to-cell signaling and what the relevant concentration of EV-, or protein-bound miRNAs would be to have a physiologically significant function. Chevillet et al. have calculated that EVs contain less than 1 copy of miRNAs per EV [57]. In sequencing experiments the small RNA cargo is analyzed in bulk, therefore there is no information whether all individual EVs are equally loaded with miRNA molecules or if there is a certain distribution of cargo into different EVs. A specific segregation of miRNA molecules may significantly raise the copy number of individual miRNAs per EV as it is proposed with low-occupancy/high-miRNA concentration distribution model indicating that rare EVs in population contain many copies of a certain miRNA [57].

As the main goal of this study, we were interested, whether the ovarian miRNA expression, secretion, or segregation into EVs are compromised by PCOS. We observed that each studied follicular compartment is affected differently by PCOS, since the miRNA profiles are dissimilar in MGC, FF and EV also in the fertile ovary. Therefore, separate pathways are affected by PCOS in MGC and in the potential recipient cells for the extracellular miRNA in the FF and EV.

Several miRNAs have been shown to be differentially expressed in the granulosa cells and/or FF between fertile and PCOS women [9,30,58,59]. In the current study, the highest number of differences in miRNA expression were detected from cellular material. At single follicle level the EV samples diverged the least between the two groups, although some differences could be detected without multiple testing adjustment. Fewer differences in EV may be caused by the more complex processing of EV samples before RNA extraction compared to MGC and FF that may affect the results.

We identified several miRNAs differentially expressed between the patient groups which have not been previously associated with PCOS but are involved in the regulation of gene expression in follicles or in other ovary-related disorders. For example, hsa-miR-224-5p [60], which was up-regulated in the MGC of PCOS women in our study, downregulates SMAD4, which is involved in the regulation of apoptosis of granulosa cells [61]. It has been shown that hsa-miR-203a-3p, hsa-miR-195-5p, hsa-miR-486-3p, and hsa-miR-484 levels are altered in the granulosa cells of women with diminished ovarian reserve [24]. Interestingly, all the four mentioned miRNAs are expressed in the MGC of our PCOS group according to the same pattern as in normal ovarian reserve (NOR) patients: hsa-miR-203-3p and hsa-miR-195-5p are both more abundant in NOR and our PCOS group, while hsa-miR-486-3p and hsa-miR-484, are less abundant in NOR as well as in our PCOS samples. PCOS women have been shown to have a slower age-related decline in antral follicle count (AFC) compared to non-PCOS patients [62] and AFC is considered to be a reliable marker for ovarian reserve evaluation [63]. Slower decline in AFC in PCOS women may also explain our miRNA expression results.

The expression differences of hsa-miR-486-5p [64] in MGC have been previously associated with PCOS compared to women undergoing IVF due to male factor infertility with same directional expression as in our study. Moreover, hsa-miR-200a-3p [34] and hsa-miR-30a-3p [32] have been previously linked to PCOS, but in other follicular compartments. In these studies, hsa-miR-200a-3p in cumulus granulosa cells and hsa-miR-30a-3p in FF were less abundant in PCOS women which is opposite to our results. Differential expression of hsa-miR-509-3-5p [30] and hsa-miR-200c-3p [30,31] in FF has been previously associated with PCOS with mutual expression direction to our results. Hsa-miR-1307-3p [65] and hsa-miR-223-3p [34] are also altered in cumulus granulosa cells of PCOS patients, but with an opposite direction to our result. These comparisons demonstrate the dependence of miRNA expression disturbances depending on the cellular environment. By the pre-ovulatory follicular stage, when the samples have been obtained, cumulus granulosa cells have differentiated from MGC and have significant dissimilarities in gene expression and post-transcriptional regulation patterns [27,66]. From EV samples hsa-miR-200c-3p [30,31], hsa-miR-17-5p [38] have been previously shown to be altered in PCOS women in line with the results of our study.

Our results clearly demonstrate that the effects of miRNA expression differences brought upon PCOS lead to different molecular outcomes depending on the investigated sample type. For example, cytokine-mediated signaling was affected in the cellular compartment and by non-EV-mediated RNA secretion, while EV-mediated signaling potentially affects the IGF1R pathways in PCOS patients. Those results emphasize the importance of studying the follicle as a system to better understand inter-cellular signaling and possible molecular disturbances in the PCOS ovary.

In conclusion, the current study proposes novel miRNAs and their regulated signaling pathways, underlying the infertility of patients with PCOS.

## 4. Materials and Methods

### 4.1. Ethics Statement

The study was approved by the Research Ethics Committee of the University of Tartu, Estonia on January 21st, 2019 with the approval number 289/M-8. Written informed consent was obtained from all participants.

### 4.2. Patients and Sample Collection

FF and MGCs were collected from women undergoing ovarian stimulation and oocyte pick-up by ovarian puncture (OPU). Ovarian hormonal stimulation was conducted according to the gonadotropin-releasing hormone (GnRH) antagonist (Cetrotide, Merck Serono, Darmstadt, Germany) protocol with the administration of recombinant follicle-stimulating hormone (Gonal-F, Merck Serono, or Puregon, Merck Sharp & Dohme Corp., Whitehouse Station, NJ, USA). All patients underwent OPU if at least two follicles were ≥18 mm in size 36 h after human chorionic gonadotropin administration (Ovitrelle, Merck Serono).

Samples were collected from two groups of women: PCOS patients (*n* = 15) and fertile women (IVF patients from couples with male factor infertility (*n* = 16) and oocyte donors (*n* = 15)). General characteristics of study participants are presented in Table 1. The PCOS group was formed according to the Rotterdam Consensus [67] with PCOM observed by ultrasound being the primary criterion for recruitment. Control group consisted of women with regular menstrual cycles, without any infertility diagnosis nor PCOM. All recruited women were <40 years of age.

FF containing all cellular material was collected from the first aspirated follicle visibly clear of blood contamination. The sample was first centrifuged 10 min at 300 g to remove whole cells. The supernatant was subsequently centrifuged 10 min at 2000× g to remove cell debris. The final cell-free FF was stored at −80 °C until further analysis. The remaining cell pellets of MGCs from the first centrifugation were lysed using QIAzol Lysis Reagent (QIAGEN, Hilden, Germany) and stored at −80 °C until RNA extraction.

### 4.3. Isolation of Extracellular Vesicles from Follicular Fluid Samples

Five hundred µL of each FF sample was concentrated to 150 µL using 10 kDa Amicon^®^ Ultra centrifugal filter units (Merck Millipore Ltd., Tullagreen, Carrigtwohill, Ireland). Commercially available size exclusion chromatography (SEC) column (qEVsingle/70 nm by Izon Sciences, UK) was used for the isolation of EVs. The column was prewashed with 10 mL filtered (0.2 µm Minisart^®^ syringe filters) Dulbecco’s phosphate-buffered saline (DPBS, Sigma^®^ Life Science, UK) and 150µl of the concentrated sample was added to the top of the column filter. After the sample had passed down, DPBS was added immediately on the top of the column filter and a total of 20 fractions, 200 µL each, were collected separately. The concentration of nanoparticles (NP) of each fraction was measured on ZetaView^®^ nanoparticle tracking analyzer (NTA, PMX 120 by Particle Metrix GmbH, Inning am Ammersee, Germany). The protein concentration of each fraction was determined with the Quick Start™ Bradford Protein Assay (Bio-Rad, California, USA) according to the manufacturer’s protocol. Based on these analyses, fraction 6-9 (800 µL) showed the presence of the highest number of particles and the least protein contamination Supplementary Figure S1). Fractions 6–9 were pooled together, concentrated with 10 kDa Amicon^®^ Ultra 2 centrifugal filter units, and used for downstream experiments.

### 4.4. Nanoparticle Tracking Analysis

The size profile and concentration of NPs/EVs in the samples were carried out using the ZetaView^®^ nanoparticle tracking analyzer. A standard of 100 nm particles (Applied Microspheres BV, Leusden, the Netherlands) was used for instrument calibration. During the analysis, standard manufacturer’s procedure was followed for NPs/EVs size distribution and concentration measurement. The size profile and concentration of NPs/EVs were measured using the scatter mode under the following settings: sensitivity 85, shutter speed 70, frame rate 30 frames per second, and the number of cycles 3. All samples were measured in triplicates. In order to minimize the inter-sample contamination, the measurement cell of the instrument was washed thoroughly using Milli-Q^®^ water, and the cell was filled with DPBS before the injection of the next sample.

### 4.5. Western Blot Analysis

Fractions 6–9 (contain follicular fluid EVs) and fractions 10–14 (contain FF proteins) were obtained by SEC as described earlier. Respective fractions were pooled and concentrated with 10 kDa Amicon^®^ Ultra-15 centrifugal filter to 300 µL. To precipitate the proteins, 100 µL of water, 400 µL of methanol (Sigma-Aldrich, Schnelldorf, Germany), and 100µl of chloroform (Lach-Ner, Neratovice, Czech Republic) were added to the concentrated sample and centrifuged for 5 min at 14,000× *g*. The top layer was removed, and proteins in the interphase were washed with 400 µL of methanol. After centrifugation, the pellet was dried, resuspended in 0.25% SDS, and the protein concentration was measured with Bradford assay. Proteins from 50µl of FF were precipitated using the same protocol. For each sample, 10 µg of protein was mixed with either non-reducing Laemmli buffer or reducing Laemmli buffer, heated for 5 min at 95 °C and separated by 12% SDS-PAGE. Proteins were transferred onto polyvinylidene difluoride membranes (Thermo Scientific, Rockford, IL, USA), and the membranes were incubated in blocking buffer (5% nonfat dry milk in PBS-Tween 0.05%) for 1 h at room temperature (RT). Subsequently, the membranes were incubated with the following primary antibodies overnight at 4 °C: mouse anti-human CD63 antibody (556019, 1:1000, BD Biosciences, San Jose, CA, USA), mouse anti-CD9 antibody (sc-59140, 1:250, Santa Cruz Biotechnology Inc., Dallas, TX, USA), mouse anti-apoA-I antibody (sc-376818, 1:1000, Santa Cruz Biotechnology Inc.), mouse anti-human CD81 antibody (555675, 1:1000, BD Biosciences), rabbit anti-Grp94 antibody (ADI-SPA-851-D, 1:1000, Enzo Life Sciences, Farmingdale, NY, USA) and rabbit anti-albumin antibody (16475-1-AP, 1:10 000, Proteintech, Chicago, IL, USA). Membranes were washed with PBS-Tween 0.05% and then incubated with either HRP-conjugated goat anti-rabbit IgG secondary antibody (G21234, 1:20 000, Invitrogen, Thermo Fisher Scientific, Eugene, OR, USA) or goat anti-mouse IgG secondary antibody (G21040, 1:20 000, Invitrogen, Thermo Fisher Scientific) for 1 h at RT. After washing the membranes with PBS-Tween 0.05% and incubating in ECL Select Western Blotting Detection Reagent solution (GE Healthcare, Chalfont St. Giles, Buckinghamshire, UK), the protein bands were visualized using ImageQuant RT ECL Imager (GE Healthcare).

### 4.6. Transmission Electron Microscopy

Fractions 6-9 (800 µL) of isolated EVs on SEC were pooled and subsequently concentrated to 150µl using Amicon^®^ Ultra 2 centrifugal filter units (10 kDa) (Merck Millipore Ltd.). A previously described method [39] was followed for *transmission electron microscopy* (TEM) analysis. A droplet from the purified EV samples was deposited on Formvar-carbon-coated 200 mesh copper grids (Agar Scientific, Essex, UK) and allowed to absorb for 20 min. The sample was fixed on a grid in 2% paraformaldehyde (Sigma-Aldrich) and 1% glutaraldehyde (Polysciences, Warrington, PA, USA), contrasted in uranyl oxalate (a mixture of 4% uranyl acetate (Polysciences) and 0.15 M oxalic acid (Sigma-Aldrich)) and embedded in a mixture of methylcellulose (Sigma-Aldrich) and uranyl acetate (Polysciences). Samples were observed with a JEM 1400 transmission electron microscope (JEOL Ltd. Tokyo, Japan) at 80 kV, and digital images were acquired with a numeric camera (Morada TEM CCD camera, Olympus, Germany).

### 4.7. RNA Extraction

miRNA extraction from isolated EVs was performed using miRNeasy Micro kit (QIAGEN) according to the user manual with the exception of 5 µg of glycogen (Thermo Scientific) added to chloroform.

Starting amount of miRNA extraction from FF was 500 µL. Extraction was performed with miRNeasy Micro kit (QIAGEN) with some modifications to the user manual [68]. Shortly, 500 µL of FF was transferred into a 15 mL tube and 5x volumes of QIAzol Lysis Reagent (QIAGEN) was added. After incubation 500 µL chloroform and 5 µg of glycogen (Thermo Scientific) were added to the tube. Following steps of RNA exactions were performed according to the miRNeasy Micro kit (QIAGEN) user manual.

Total RNA from cells was extracted with miRNeasy Mini kit (QIAGEN). In addition, small fraction RNA (≤200 nucleotides) was separated by RNeasy Mini Elute Cleanup Kit (QIAGEN). Both total and small RNA extraction were performed according to the user manual.

The quality and concentration of cellular RNA samples was evaluated on Agilent 2100 Bioanalyzer (Agilent Technologies, Waldbronn, Germany).

### 4.8. Small RNA Library Preparation and Sequencing

Small RNA libraries were prepared with QIAseq miRNA Library Kit (QIAGEN) according to the manufacturer’s protocol. Starting amount of RNA in library preparation was 10 ng from cellular small RNA fraction and 5 µL of RNA from EV and FF samples. Final libraries were separated and excised from 5% TBE gels (Bio-Rad Laboratories) after staining with 1X SYBR Gold stain (Thermo Fisher Scientific). Gel pieces containing the miRNA libraries were crushed with pellet pestles (Fisher Scientific). 300 µL RNase free water (Thermo Fisher Scientific) was added to the gel debris and rotated for 2 h at RT to elute miRNA libraries. Eluate and gel debris were transferred to the Spin X centrifuge tube filter (Merck, Darmstadt, Germany) and centrifuged 2 min at 16,000 g. Thereafter 2 µL glycogen (Thermo Scientific), 30 µL 3 M NaOAc (Thermo Fisher Scientific), 1 µL 0.1x Pellet Paint (Merck) and 975 µL of cold 100% ethanol (Naxo, Tartu, Estonia) were added to the eluate, and centrifuged for 20 min at 20,000× *g* at 4 °C. Pellet was washed with 500 µL of 70% ethanol and centrifuged for 2 min at 20,000× g. The final libraries were resuspended in 7 µL of resuspension buffer (PerkinElmer, Massachusetts, USA). The size of libraries was estimated with Agilent DNA High Sensitivity chips on the Agilent 2100 Bioanalyzer system (Agilent Technologies). Library concentrations were measured using Qubit High Sensitivity Assay kit (Thermo Fisher Scientific) before pooling in equimolar amounts. Single-end sequencing of 75 bp length was performed on NextSeq 500 platform with NextSeq 500/550 High Output Kit v2.5 (Illumina, San Diego, CA, USA).

### 4.9. cDNA Synthesis and RT-qPCR

For the validation of miRNA expression levels cDNA was synthesized using miRCURY LNA RT Kit (QIAGEN) from 30 ng of cellular small RNA fraction or 5 µL of extracted small RNA fraction from FF and EV samples.

The RT-qPCR analysis was carried out on LightCycler 480 instrument (Roche, Basel, Switzerland). For the detection of miRNA expression miRCURY LNA SYBR Green (QIAGEN) was used according to the user manual. The specificity of amplified PCR products was determined by melt curve analysis. miRCURY LNA miRNA PCR Assay primers were used in all reactions (QIAGEN).

### 4.10. Data Analysis and Statistics

#### 4.10.1. miRNA Sequencing Analysis

Raw FASTQ files were quality-filtered with Trimmomatic v 0.39 [69] with the options of SLIDINGWINDOW:2:20. Adapter sequences (3’adapter AACTGTAGGCACCATCAAT and 5´ adapter GTTCAGAGTTCTACAGTCCGACGATC) were removed and reads below 17 nucleotides in length were discarded and the remaining filtered and trimmed reads were counted and mapped to the primary assembly of human genome GRCh38 using miRDeep2 with standard settings [70].

Count tables from individual samples were merged using edgeR package v.3.28.1 [71] and formed count matrix was used as input for DESeq2 v.1.26.0 [72] in R version 3.6.3 [73] for differential gene expression analysis between groups with standard options. miRNAs expressed at low level were removed from analysis: cut-off was set at ≥5 raw reads in 50% of samples. For visualization purposes, variance stabilizing transformation of data was performed with option blind = FALSE.

The statistical significance cut-off for differentially expressed miRNAs in DESeq2 analysis was set at false discovery rate (FDR) <0.05 in case of comparing three tissue types in oocyte donor samples. Cut-off for statistical significance was set at FDR <0.1 when comparing patient groups.

#### 4.10.2. RT-qPCR Data Analysis

miRNA expression levels in cellular fraction were normalized for U6 snRNA and hsa-miR-132-3p. Endogenous control for FF and EV samples was hsa-miR-16-5p. All normalizations were performed according to the ΔΔCt method of relative quantification [74]. Statistical significance was calculated by two-tailed Student’s t-test in Microsoft Office Excel 2017. Statistical significance level was set at *p* < 0.05.

#### 4.10.3. miRNA Target Prediction, Gene Ontology and Over-Representation Analysis

Novel miRNA targets were predicted with miRDB [75] custom prediction tool. Obtained miRNA targets list was an input for gene enrichment analysis with g:Profiler [76], using g:GOst functional profiling tool where significance threshold was set at FDR <0.05. Results were visualized with REVIGO [77].

For annotated miRNAs the lists of statistically significant differentially expressed miRNA lists were used as input to miRNA Enrichment Analysis and Annotation Tool (miEAA), that performs miRNA target prediction and over-representation analysis of gene ontology terms simultaneously by combining linked external databases [78]. Over-representation analysis was performed for Reactome Pathways via miRPathDB [79] for each up- and down-regulated miRNA list separately. Background list was created from all detected miRNAs in our small RNA sequencing dataset. Pathways targeted by >50% of miRNAs (minimum 3) in each list with Benjamini-Hochberg FDR < 0.05 are reported.

Similarly, miEAA was used to analyze Reactome Pathway over-representation for all miRNAs observed exclusively in FF or in EV samples.

#### 4.10.4. Novel miRNA Candidate Filtering

Predicted novel miRNAs were filtered with a cut-off of miRDeep2 score >1. Remaining potential novel miRNA candidates were aligned against human transcriptome with NCBI nucleotide BLAST (https://blast.ncbi.nlm.nih.gov/Blast.cgi), discarded if the sequences overlapped with a coding region of an annotated gene, demonstrated high similarity to other know miRNAs, were detected only in one sample or with the average occurrence in positive samples <10 raw counts. miRNA sequences with similar seed region to the potential novel miRNA were obtained from miRBase v22.1 and visualized in Jalview 2.11.1.0 [80].

#### 4.10.5. EV Size Profile and Concentration

To test if the sample means of the EV size profiles are non-normally distributed 1000 samples of 1000 EVs were drawn from the NTA data of PCOS and donors’ group, their means calculated and tested using Shapiro–Wilk test. Student’s t-test was then used to test if the difference between the size profile means is statistically significant. The behavior of *p*-values with smaller sample size was further analyzed by drawing 1000 random samples of size 100, 1000, 2000, and 5000 EVs each and plotting the *p*-value histograms (Supplementary Figure S2). The difference in EV concentrations was tested using two-tailed Student’s *t*-test and a *p*-value <0.05 was considered statistically significant.

#### 4.10.6. Data Availability

The datasets generated for this study can be found in the Gene Expression Omnibus repository (GSE157037).

## 5. Conclusions

The current study proposes novel signaling pathways underlying the infertility of patients with PCOS. We demonstrate that the follicular environment is affected by the PCOS differently depending on the studied compartment, i.e., MGC, EV, and FF, indicating potential changes in intercellular communication in the ovaries of these patients. We predict that alterations in cellular miRNA expression levels lead to changes in estrogen receptor signaling and the dysregulation of transcription and apoptosis. EV-mediated miRNA signalization potentially affects IGF1R pathways in the recipient cells.

## Figures and Tables

**Table 1:** General characteristics of study participants by used method.

		Age (Mean, Years)	SD (Years)	BMI (Mean, kg/m^2^)	SD (kg/m^2^)
**Extracellular vesicle characterization:**					
PCOS	*n* = 15	32.7	4.3	23.5	3.1
Oocyte donors	*n* = 15	25.8	3.2	22.3	2.8
*p*-value		<0.001		0.313	
**Small RNA sequencing:**					
PCOS	*n* = 7	34	4.8	22.7	2.6
Oocyte donors	*n* = 8	26.9	2.2	22.7	3.6
*p*-value		0.002		0.999	
**miRNA expression validation (RT-qPCR):**					
PCOS	*n* = 15	32.7	4.3	23.5	3.1
Control group	*n* = 16	33	4	23	3
*p*-value		0.626		0.831	

**Table 2:** Differentially expressed miRNAs between PCOS patients and oocyte donor samples selected for RT-qPCR validation.

**A) MGC PCOS vs. donors:**		
**miRNA**	**Log_2_ Fold Change**	**miRNA role in ovary**
hsa-miR-205-5p	4.82	Expression is upregulated in MI oocytes upon IGF-1 treatment [22]. Upregulated in ovarian cancer (OC) cells compared to control group and is associated with poor survival rates. Proposed miRNA targets are SMAD4 and PTEN [23].
hsa-miR-203a-3p	3.56	Expression levels are higher in granulosa cells of young women with normal ovarian reserve compared to young women with diminished ovarian reserve [24].
hsa-miR-196a-5p	2.49	Detectable in bovine granulosa cells at day 3 but not at day 7 of the estrous cycle [25].
hsa-let-7c-5p	1.45	Expressed higher in human CGC compared to MGC cells. miRNA expression is decreased in granulosa cells of early and progressive atretic follicles and in case of premature ovarian failure syndrome (measured from plasma) [26].
hsa-miR-181a-5p	−1.56	Expressed higher in human CGC compared to MGC [27]. In mouse granulosa cells miR-181a-5p targets ACVR2A (Activin Receptor IIA) and inhibits granulosa cell proliferation [28]. In oxidative stress conditions miRNA expression is upregulated in mouse granulosa cells and mediates granulosa cell apoptosis [29].
**B) FF PCOS vs. donors:**		
**miRNA**	**Log_2_ Fold Change**	**miRNA role in ovary**
hsa-miR-509-3-5p	2.80	Expression is higher in FF of PCOS patients compared to controls [30].
hsa-miR-200c-3p	2.26	Expression is higher in granulosa cells [31] as well as in FF samples [32] of PCOS patients compared to control group.
hsa-miR-223-3p	2.02	EVs obtained from FF show expression of hsa-miR-223-3p [33]. miRNA expression is decreased in cumulus cells of PCOS patients [34].
hsa-miR-1908-5p	−1.48	Low expression predicts poor prognosis for ovarian cancer [35].
hsa-miR-146a-5p	−1.81	Expression is higher in human MGC samples compared to CGC [36].
**C) EV PCOS vs. donors:**		
**miRNA**	**Log_2_ Fold Change**	**miRNA role in ovary**
hsa-miR-200c-3p	2.77	Expression is higher in granulosa cells samples [31] as well as in FF [32] obtained from PCOS patients compared to control group.
hsa-miR-100-5p	1.42	Associated with cell proliferation regulation [37]. Downregulated in young women with diminished ovarian reserve compared to normal ovarian reserve [37].
hsa-miR-17-5p	−1.55	Expression is downregulated in granulosa cells and FF of PCOS women compared to controls [38]. miRNA expression is detected in EVs obtained from FF [33].

## Supplementary Materials

Supplementary materials can be found at https://www.mdpi.com/1422-0067/21/24/9550/s1.

## Abbreviations

AFC	Antral follicle count
CGC	Cumulus granulosa cells
COC	Cumulus oocyte complex
DE	Differential expression
ESR	Estrogen receptors
EV	Extracellular vesicles
FDR	False discovery rate
FF	Follicular fluid
MGC	Mural granulosa cells
NOR	Normal ovarian reserve
NP	Nanoparticles
NTA	Nanoparticle tracking analyzer
PCOM	Polycystic ovarian morphology
PCOS	Polycystic ovary syndrome
RBP	Ribo-protein complexes
SEC	Size exclusion chromatography
TEM	Transmission electron microscopy
WB	Western blot
