# Supplementary material for: Cellular, Extracellular and Extracellular Vesicular miRNA Profiles of Pre-Ovulatory Follicles Indicate Signaling Disturbances in Polycystic Ovaries
Source: Int J Mol Sci. 2020 Dec 15;21(24):9550. doi: 10.3390/ijms21249550 (PMC7765449; doi:10.3390/ijms21249550)
Supplement: Supplementary file 1 [file ijms-21-09550-s001.zip › Supplementary_Results_IJMS.docx]

Supplementary Material

#### Specification of isolated nanoparticles by qEVsingle size exclusion chromatography column

# qEVsingle® size exclusion chromatography column (SEC) was used to isolate EVs, and a total of 20 fractions were collected (each 200 µl) as mentioned in materials and methods section “Isolation of extracellular vesicles from follicular fluid”. The EV concentration was determined using ZetaView® nanoparticle tracking analyser (NTA). NTA analysis revealed that fraction 6-9 contains the highest number of nanoparticles/EVs (SU Figure 1) and no significant protein contamination. The fractions 1-5 contained no particles (void volume), whereas fractions from 10-20 contained fewer nanoparticles and relatively higher protein concentration. Based on these results, fraction 6-9 were pooled together, concentrated with Amicon® Ultra 2 centrifugal filter units (10 kDa), and were used for downstream experiments.

#
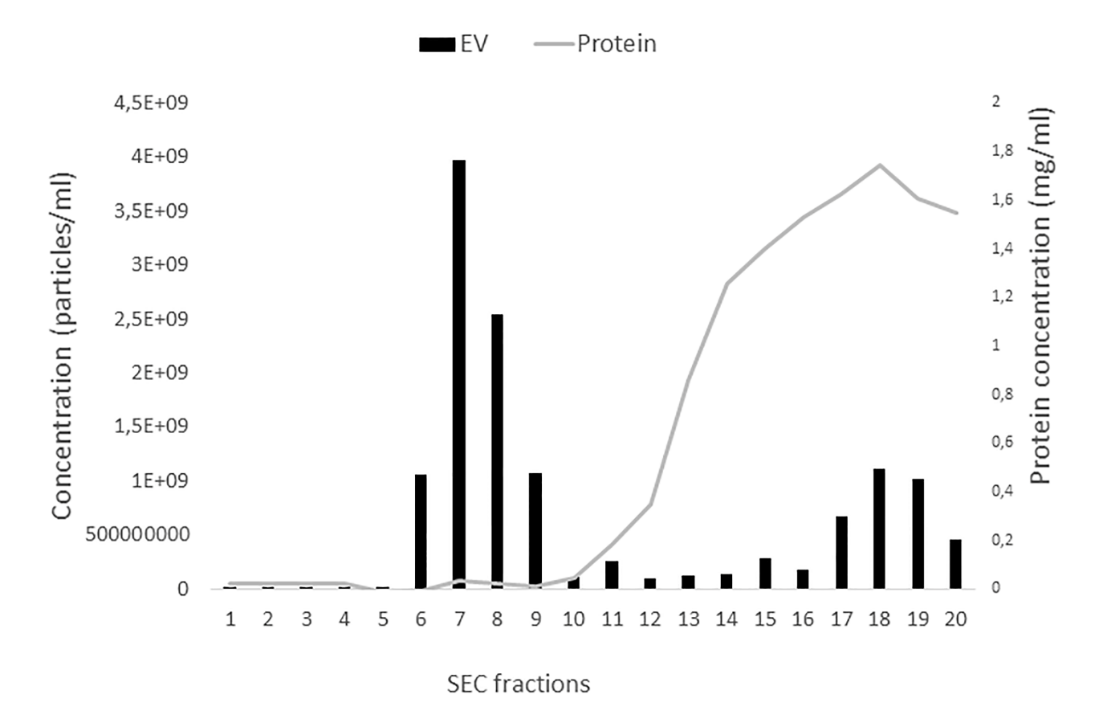


**Supplementary Figure S1**. Evaluating the profile of nanoparticles/EVs and protein concentration of fractions isolated on qEVsingle® column. Fraction 6-9 (EV fraction) contained the highest number of nanoparticles and relatively low protein contamination. Particle concentration was analyzed using ZetaView® nanoparticle tracking analyzer (NTA), and the protein concentration was measured using Quick Start™ Bradford Protein Assay


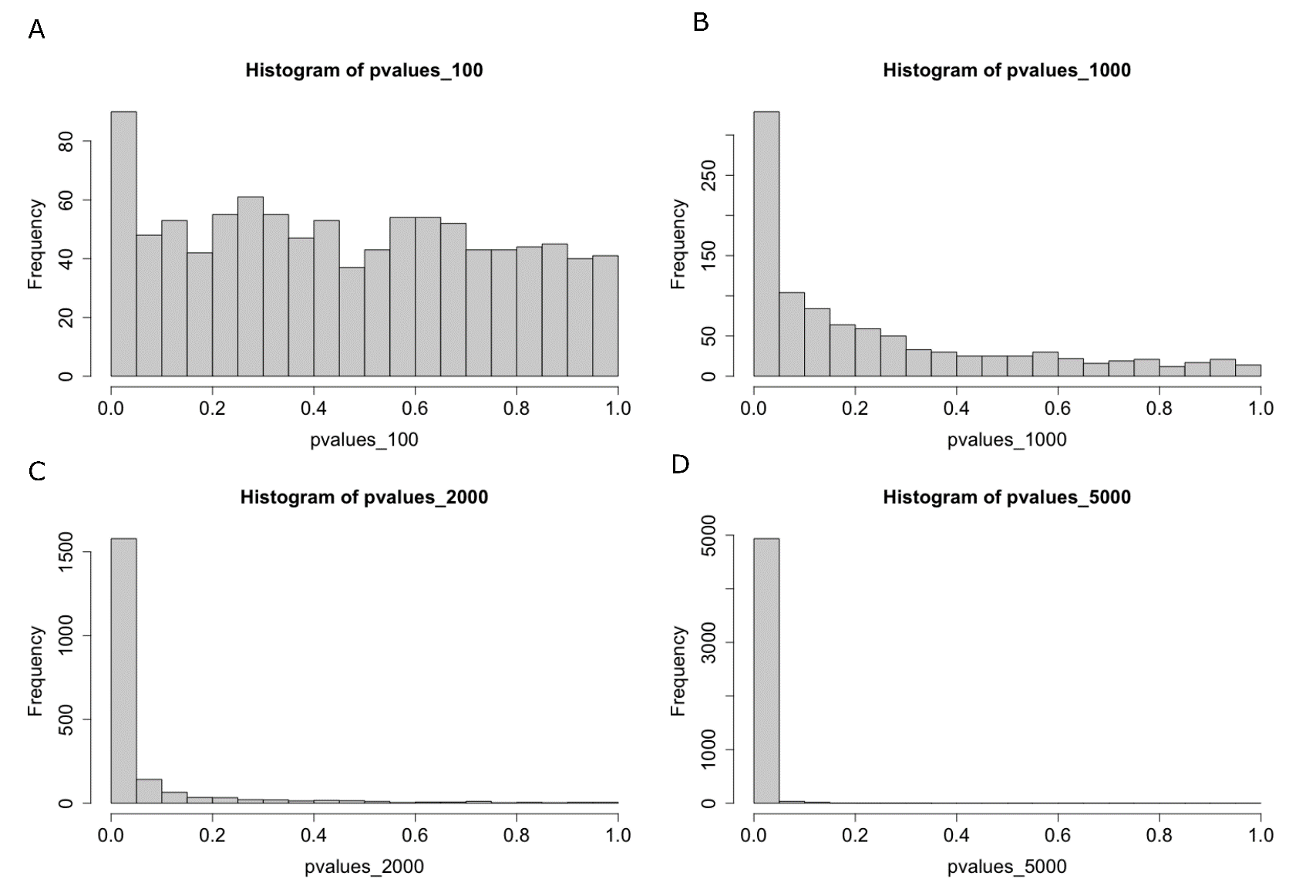


**Supplementary Figure S2** Histograms of p-values with 1 000 samples drawn from the EV size distributions with sizes of (**A**) 100, (**B**) 1 000, (**C**) 2 000, and (**D**) 5 000. P-values indicating statistical significance are clearly overrepresented even with the sample size of 100.

**Small RNA sequencing read size distribution in granulosa cells, cell-free follicular fluid and extracellular vesicles**

**
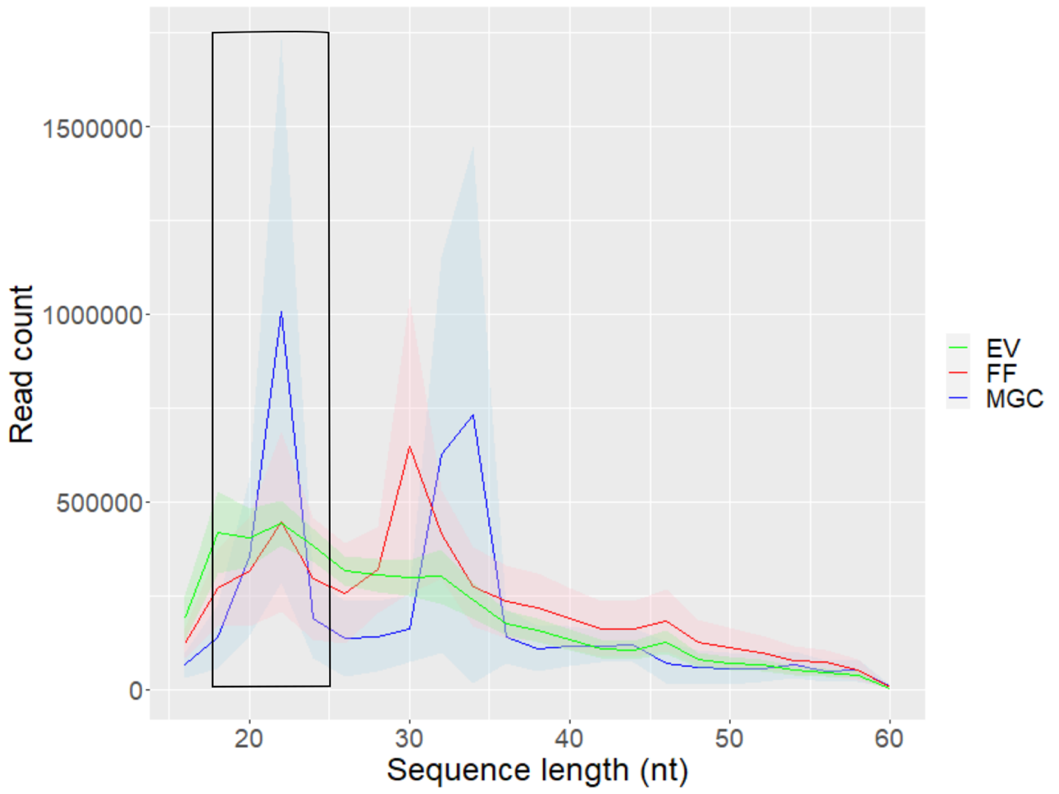
**

**Supplementary Figure S3**. Average length distribution of sequenced RNA molecules from all sample types (mean ±SD). Read length corresponding to miRNAs is marked by black box. EV-extracellular vesicles, FF-follicular fluid, MGC-granulosa cells.

**Differentially Expressed miRNAs between sample types in the healthy follicle**

**
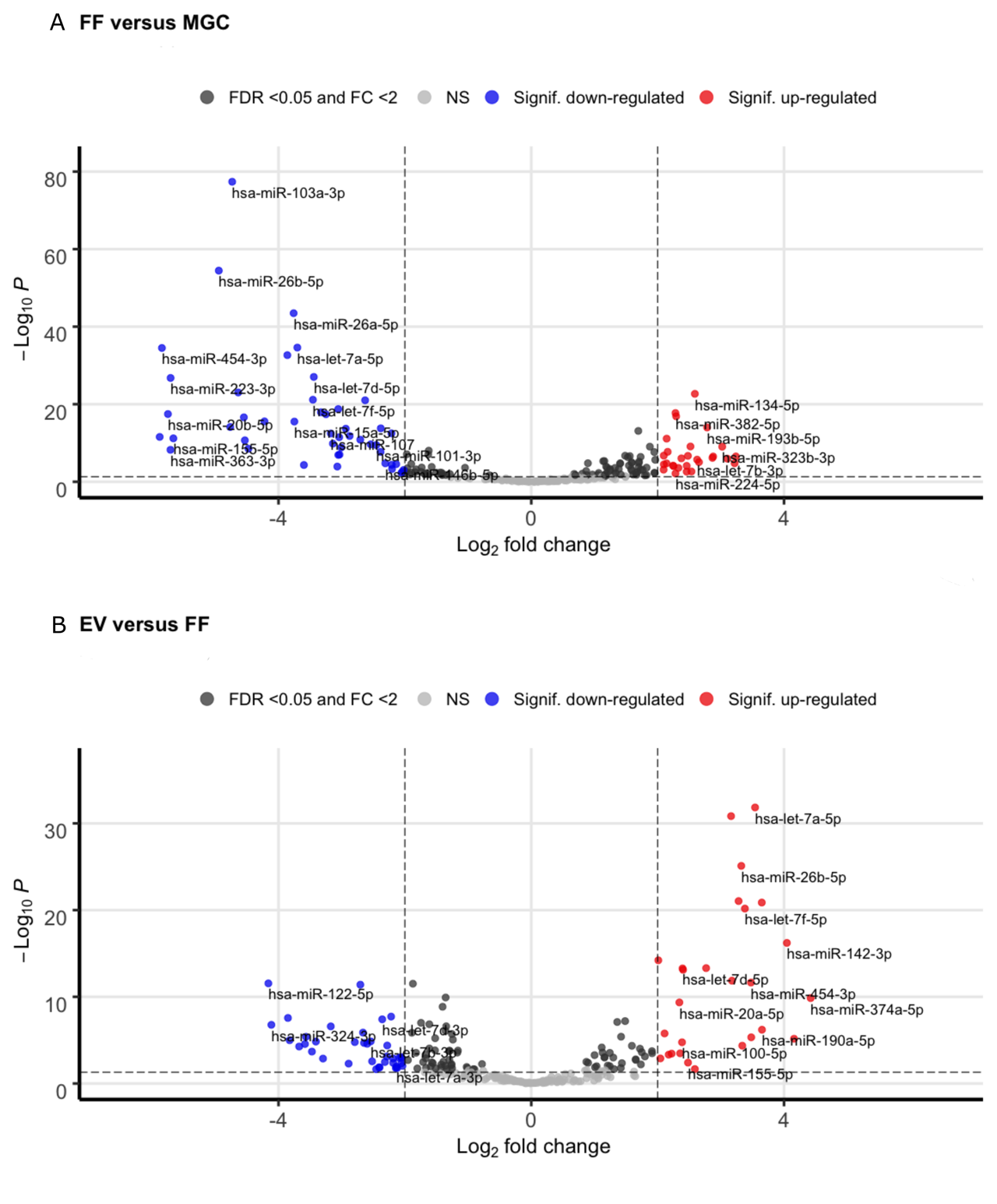
**

**Supplementary Figure S4.** Differentially expressed miRNAs between sample types. (**A**) Cell-free follicular fluid (FF) versus granulosa cells (MGC). (**B**) Extracellular vesicles of the follicular fluid (EV) versus FF.

**Validation of sequencing results by RT-qPCR**


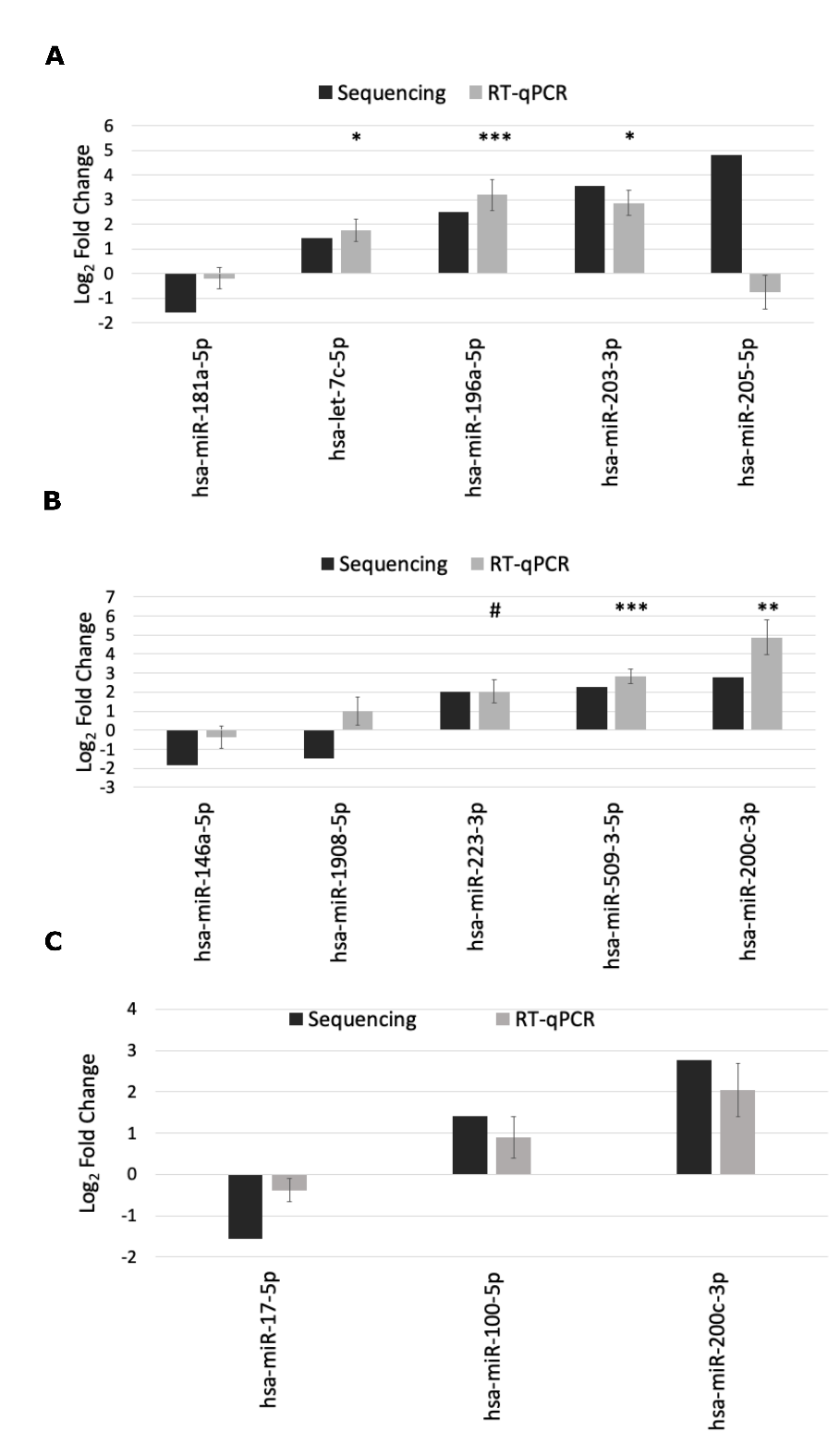


**Supplementary Figure S5.** Comparison of RNA sequencing and RT-qPCR results of miRNA expression levels between PCOS and control samples. (**A**) Granulosa cell samples (MGC). (**B**) Cell-free follicular fluid (FF). (**C**) Extracellular vesicles of the follicular fluid (EV). Results are displayed as a mean of fold change ±SEM on log­_2_ scale (#=0.051, *p < 0.05, **<0.01 ***p < 0.001, Student’s t-test

**Functions of the predicted targets for the novel miRNA sequence**

**Supplementary Figure S6.** Treemap of functional enrichment analysis results of the novel miRNA targets. Gene ontology terms reflecting enriched biological processes are categorized into superclusters depicted in different colours.
